# Letter from Edmund Andrews, M.D.

**Published:** 1867-10

**Authors:** Edmund Andrews

**Affiliations:** London


LONDON, July 25, 1867.

TO THE EDITOR OF THE EXAMINER:

The state of the medical profession in London is worthy of both praise and blame. In the way of praise, it may be said that their system of education and examinations insures that every man who enters the profession shall have at least a passable degree of knowledge. It is impossible for a perfect ignoramus to get a diploma or to become recognized as a member of the profession. In some things, however, they need reforms. The Examiners here are not the members of the faculties who give the instruction, but a Board appointed by the Government. They are venerable old fellows, the very youngest of whom is 60 years of age. As a matter of course, their examinations smack strongly of the last century, instead of being up with the times. The professors in the medical colleges complain that they have to give two sets of instructions to their pupils, one to inform them of the answers which their ancient Board will require, and the other to give them the truth. Meanwhile, the members of the Board disdain to learn anything, because they are too old, and they will not resign because their position brings them two or three thousand dollars a year, and so the evil is perpetuated without end.

The London surgeons are very deficient in their attention to hospital ventilation. I have not been able to find more than one of these buildings which was ventilated with anywhere near the completeness which we have been accustomed to attain in

Mercy Hospital. The one well ventilated institution here is Middlesex Hospital. I was much interested to observe a fact, which coincides with my experience in Chicago. In Middlesex, exactly as in Mercy Hospital, deaths from pyæmia almost never occur. That part of the mortality of surgery is practically abolished, while in other London hospitals, where ventilation is bad, pyæmia and other kindred results of foul air, cause from ten to twenty-five per cent of the deaths in surgical cases. Most of the surgeons here admit the effect of the ill-ventilation in causing pyæmia, but they are thick-headed, and inefficient in taking action upon it. I have seen them give all sorts of orders about medicine, food, drink, applications, etc., etc., "tithing mint, anise, and cummin," with great precision, but never heard one of them order the windows to be opened, though their patients were dying under their eyes for want of pure air. The fact is, some of the great London hospitals are a curse to the community, for they kill more patients by pæmia, erysipelas, and other diseases generated by close air, than all their surgeons, or anybody else, can cure by the skill of their operations.

Some valuable improvements, however, are being attempted in this country; how far they will stand the test of experience remains to be seen. The surgeons in Glasgow are at present rejoicing in the use of carbolic acid. This substance resembles creosote in its properties. In case of compound fractures and other bad wounds, lint is dipped into a strong solution of the acid and stuffed into the wound until it is full. After a day or two it is removed and a new filling put in the same way, but with a weaker solution. It is claimed that by this method *all suppuration* is prevented, as well as all decomposition of tissue or secretions, that the wounds fill rapidly with new flesh without any pus, and that pyæmia, suppurative exhaustion, and erysipelas are prevented. One of the surgeons at Guy's Hospital showed me a compound fracture extending into the knee-joint, which he treated in that way. The joint never suppurated, nor even inflamed in the least, and healed with rapidity. He stuffed a wound of a compound fracture of the leg, with the preparation, in my presence. The next day, I visited the patient, and found that he had suffered much pain, and looked haggard and worn. I think he appeared worse than men usually do who are treated in a milder way. However, if it is a settled thing that an English hospital must not be ventilated, I think carbolic acid may be a good thing, as being, next after fresh air, the best preventive of pyæmia. It is powerfully antiseptic, and if it will prevent suppuration, it may be very valuable in cases of hectic exhaustion.

The English hospital officers seem generally to be totally ignorant of the value of adhesive-strap extention in fractures. They also are equally blind to the advantages of the single inclined plane for the fractures of the thigh, whereby the irritation of the perineal band is avoided. They generally use the old-fashioned long splint and perineal band.

There is one appendage to every hospital here which we would do well to use more frequently in America, and that is the water-bed. This, as your readers know, is a chest, or tight bedstead of metal or plank, filled with water. The top is covered with a loose rubber cloth, floating on the water, and made fast and tight at the edges. Patients who have paralysis, or other troubles, which predispose to bed-sores, are laid in these couches, where they float, as it were, in the water, and the pressure being equable on all parts, the bed-sores are prevented.

The lecture rooms in the hospitals and medical colleges here are most uncomfortable places. The seats are usually simple boards, only nine inches wide, and without any backs. The patience of the students is most praiseworthy, especially as the ecturers are often very dull in the manner of delivery.

Resections of the knee are extensively practiced, even in children. The surgeons deny that it will prevent the limb from growing, provided you do not remove the whole of the epiphysis. The practice here is very often to amputate or resect inflamed knees, in cases where a Chicago surgeon would save the limb and effect a cure. They operate in the first stages, while the disease is yet a simple inflammation, without suppuration or caries; and while, according to American experience, the knee is perfectly curable. In justification of this practice, they say that the cure, if accomplished at all, would be excessively slow, and, therefore, the hospitals could not keep the patients long enough. They would go out and run about on their limbs, and exasperate the disease to actual caries; therefore they think it better to operate at once. The real fact is this: They treat their patients only by medicines, local applications, and rest in the foul air of the wards, and find that they die. As a general rule, they are grossly ignorant of the fact that adhesive-strap extension, and pure air will cure the patient without operation. So they take the easiest course, and cut out the joint, or cut off the limb, and thus end the matter, and frequently kill the patient.

Not long since I visited the Dreadnought Hospital. This is for seamen of all nations, and is supported by private charity. The building consists of an old 120 gun ship-of-war, moored in the Thames. It is well cared for, so far as it can be, but the surgeons complain that, being built for battle, and not for the sick, it is impossible to ventilate it, and that, in consequence, they are scourged severely with pyæmia. They add, also, the curious fact, that every patient who comes in has an attack of bronchitis. As usual, in marine hospitals, the venereal diseases are a prominent pathological element.

Apropos of venereal diseases, they are raising again the question here, whether mercurials shall be given in syphilis. Dr. DRYSDALE leads off in the negative, with a pamphlet, and has some followers.

On the whole, the impression remains on my mind that the London surgeons are, as a class, sound and able men. They are not brilliant, nor showy, but they are solid, and generally not disposed to stretch the truth, and use deceit, for the sake of exaggerating some supposed discovery.

They are, however, slow, and, in some respects, behind the times, and are culpable for not bringing to bear on their patients all the knowledge which they might easily possess.

EDMUND ANDREWS, M.D.

